# How I treat HER2-positive early breast cancer: how long adjuvant trastuzumab is needed?

**DOI:** 10.1016/j.esmoop.2022.100428

**Published:** 2022-03-07

**Authors:** S. Morganti, G. Bianchini, A. Giordano, M. Giuliano, G. Curigliano, C. Criscitiello

**Affiliations:** 1 Division of Early Drug Development, European Institute of Oncology IRCCS, Milan, Italy; 2 Department of Oncology and Hematology, University of Milan, Milan, Italy; 3 Dana-Farber Cancer Institute, Boston, USA; 4 Department of Medical Oncology, IRCCS Ospedale San Raffaele, Milan, Italy; 5 Department of Clinical Medicine and Surgery, University of Naples Federico II, Naples, Italy

**Keywords:** early breast cancer, trastuzumab, HER2, de-escalation, 6-month

## Abstract

Since its first approval in 2006, 1 year of adjuvant trastuzumab has been the standard of care for early-stage HER2-positive breast cancer. Nevertheless, the optimal duration of adjuvant trastuzumab was uncertain, and the standard 12-month duration has been questioned by a number of different trials. Although most of these studies were formally negative, a patient-level meta-analysis presented at the 2021 European Society for Medical Oncology (ESMO) meeting first showed the non-inferiority of 6-month trastuzumab. Through this review, we sought to take a closer look at the meta-analysis and the included trials to explain why we believe that non-inferiority should be interpreted with caution. Indeed, here we underline how the meta-analysis’ results were mainly driven by the PERSEPHONE study, an old trial that tested non-standard chemo-trastuzumab regimens in a relatively low-risk population with doubtful endpoints. In summary, considering all the limitations of this analysis and the increasing use of effective anthracycline-free de-escalation strategies, we are convinced that 1-year trastuzumab should remain the standard of care.

## Highlights

• The standard 1-year duration of adjuvant trastuzumab has been questioned by a number of trials.
• At ESMO 2021, the meta-analysis by Earl et al. showed that 6-month adjuvant trastuzumab is not inferior to 12-month.
• The PERSEPHONE trial was the main driver of the meta-analysis’ results, but it has several limitations.
• Alternative anthracycline-free de-escalation strategies proved to be effective for HER2+ early breast cancer patients.
• 12-month adjuvant trastuzumab should remain the standard of care.

## Introduction

Following the results of four major adjuvant trials (NCCTG N9831, NSABP B-31, HERA and BCIRG-006),^1^, ^2^, ^3^ administration of adjuvant trastuzumab for 1 year concurrently to chemotherapy became the standard of care for patients with HER2-positive early breast cancer (eBC). Although the addition of pertuzumab recently proved to increase outcomes in patients at high risk,^4^ trastuzumab monotherapy is still the standard of care for low-to intermediate-risk patients. In this context, the choice of a 12-month trastuzumab duration was somewhat empirical, leading in the last 20 years to several attempts to evaluate optimal trastuzumab duration. Two years of trastuzumab are clearly not superior,^5^ and shorter durations (e.g. 6 months or less) mostly failed to prove the non-inferiority. The PERSEPHONE study,^6^ the largest of these de-escalation studies, was the only one that succeeded to prove the non-inferiority of 6 versus 12 months of trastuzumab.

At the European Society for Medical Oncology (ESMO) 2021 meeting, Earl and colleagues presented the results of a patient-level meta-analysis that pooled data from five of these studies, namely the SHORT-HER, SOLD, HORG, PHARE and PERSEPHONE trials, and concluded that 6-month trastuzumab might be considered non-inferior to the standard 12-month duration.^7^ Nevertheless, these findings have been greeted with some skepticism. No subgroup analyses have been presented, and included trials have several limitations. Indeed, many questions remained unanswered at that moment, and we believe that this ‘one-size-fits-all’ finding should be interpreted with great caution.

### Adjuvant trastuzumab for less than 1 year: the background

To properly interpret the results of this meta-analysis, a close look at each individual study is needed. Based on the promising results of the small FinHER study,^8^ which showed that 9 weeks of trastuzumab might be enough to significantly decrease the risk of relapse, the SHORT-HER^9^ and the SOLD^10^ trials investigated whether 9 weeks of trastuzumab concomitant to chemotherapy was not inferior to the standard 12 months. In both studies, the primary endpoint was disease-free survival (DFS), with a similar non-inferiority hazard ratio (HR) boundary set at 1.29 and 1.3 in the SHORT-HER and SOLD studies, respectively. The SHORT-HER study enrolled 1254 patients across Italy. After a median follow-up of 6 years and 200 events, 5-year DFS was 88% in the standard arm versus 85% in the experimental arm, with a HR of 1.13 [90% confidence interval (CI) 0.89-1.42] that crossed the non-inferiority margin. More importantly, in the high-risk group, 9 weeks of trastuzumab was statistically significantly inferior to 12 months. The SOLD trial, which enrolled 2174 patients, provided similar results, with 90.5% and 88% of patients remaining disease free at 5 years in the standard and experimental arms, respectively. This trial also failed to demonstrate the non-inferiority of a 9-week regimen, with an observed HR of 1.39 (90% CI 1.12-1.72) crossing the non-inferiority threshold. Of note, the lower CI higher than 1 strongly suggests a significant inferiority of the 9-week regimen.

In the HORG, PHARE and PERSEPHONE studies, 12 months of trastuzumab was compared to 6 months.

The HORG study was the smallest, enrolling 481 patients.^11^ The primary endpoint of 3-year DFS was 95.7% for patients receiving the standard 12-month trastuzumab versus 93.3% for patients who received only 6 months. Observed HR was 1.57 (95% CI 0.86-2.10; *P* = 0.137), which crossed the non-inferiority cut-off of 1.53. Notably, in this trial the same sequential anthracycline/taxane regimen was given to all patients, and trastuzumab was administered concurrently to chemotherapy. The PHARE^12^ and PERSEPHONE^6^ trials were instead two similar large trials, both derived from academic efforts, whose results were published simultaneously in 2019. The PHARE enrolled 3308 patients across France, whereas the PERSEPHONE included 4089 patients from the United Kingdom. The non-inferiority margin was set at 1.15 in the PHARE and 1.32 in the PERSEPHONE trial. Of note, while the former was prespecified and calculated assuming a 2% difference in 2-year DFS between the two arms, the latter was calculated at the time of analysis, based on the 4-year DFS observed in the standard group and considering a non-inferiority margin of 3%.

After a median follow-up of 7.5 years and 704 events, the adjusted HR for DFS observed in the PHARE trial was 1.08 (95% CI 0.93-1.25; *P* = 0.39). Similarly, with a median follow-up of 5.4 years and 512 events, a 4-year DFS HR of 1.07 (90% CI 0.93-1.24; *P* *=* 0.011) was observed in the PERSEPHONE trial. Despite being almost overlapping, these values led to opposite conclusions due to the different non-inferiority cut-off set in each trial, with Earl and colleagues (PERSEPHONE) claiming for the non-inferiority of 6-month trastuzumab, while Pivot and colleagues (PHARE) were unable to prove it. In both trials, heterogenous chemotherapy regimens were given and trastuzumab was administered both concomitantly and sequentially.

### 12-month versus a shorter duration: a closer look into the meta-analysis

A total of 11 389 patients were included in the recently presented patient-level meta-analysis.^7^ The analysis was conducted on the intention-to-treat population, and the primary endpoint was invasive disease-free survival (IDFS). A non-inferiority absolute 2% margin was prespecified to calculate the non-inferiority HR limit, which was set as 1.19 for all five trials, and 1.20 for trials comparing 12 versus 6 months. Considering all trials combined, the analysis failed to show non-inferiority for the shorter duration (9 weeks and 6 months combined) versus 12 months, as the upper limit of the credibility interval (CrI) crossed the non-inferiority margin [adjusted HR 1.14 (95% CrI 0.88-1.47, *P* = 0.37)] with a 5-year IDFS of 88.46% versus 86.87% in the 12-month versus short duration, respectively. Demonstration of non-inferiority was instead met combining the 12- versus 6-month duration in the PERSEPHONE, PHARE and HORG trials, with a 5-year IDFS of 89.26% versus 88.56% in the 12- versus 6-month regimen, respectively, and an adjusted HR of 1.07 (95% CrI 0.98-1.17, *P* = 0.02). Of note, results were mainly driven by the PERSEPHONE data, which included more than half of the patients included in the 12- versus 6-month meta-analysis and that was the only formally positive trial. In this regard, a closer look into the population enrolled in the PERSEPHONE trial and its findings is worth it.

### The PERSEPHONE trial reviewed: features leading to the non-inferiority

In the PERSEPHONE trial, 4088 patients were randomized in an 8-year timeframe, since October 2007 to July 2015. All patients with HER2+ eBC were eligible, regardless of tumor stage. Sixty-nine percent enrolled patients had estrogen receptor (ER)-positive disease, and 59% had node-negative disease. Eighty-five percent of patients received chemotherapy in the adjuvant setting, but only 47% had concurrent administration of trastuzumab with chemotherapy. Of note, 90% of patients received an anthracycline-based chemotherapy regimen. Four-year DFS was 89.8% and 89.4% in the 12- and 6-month groups, respectively.^6^

Notably, this population is at a significantly lower risk than patients included in large randomized trials investigating adjuvant trastuzumab. Only 282 patients had node-negative eBC in the N9831 study (14.5%), and none in the B-31.^1^ In the BCIRG 006 trial, 29% of patients were node-negative.^13^ Indeed, the 4-year DFS observed with 12 months of trastuzumab in the PERSEPHONE study (89.8%) was higher than that estimated in the statistical analysis plan (80%), and that observed in both the B-31/N9831 joint analysis^14^ (85.7%) and the BCIRG 006 study^13^ (84% for docetaxel-carboplatin-trastuzumab, 86% for doxorubicin-cyclophosphamide followed by docetaxel-trastuzumab). In such a low-risk population, events other than distant recurrences or cancer-related deaths may represent a not-negligible percentage of disease survival events. As shown in the joint analysis of the NCCTG N9831 and NSABP B-31 trials,^14^ trastuzumab has a major impact on risk-related events like distant recurrences and breast cancer deaths, whereas the efficacy in preventing risk-unrelated events including local recurrences or contralateral breast cancers is low, and obviously null in preventing unrelated events like second primaries and non-breast cancer deaths. Therefore, a longer follow-up, evaluation of distant recurrence-free interval and a subgroup analysis for patients at higher risk of recurrence may help clarifying whether six additional months of trastuzumab are actually beneficial in the PERSEPHONE trial.

Earl and colleagues also presented a subgroup analysis for the preplanned stratification groups.^6^ Notably, the interaction test in the DFS analysis showed heterogeneity for both type and timing of chemotherapy. Patients receiving taxanes without anthracyclines seemed to derive a significantly higher benefit from 12-month trastuzumab, even though the number of patients in this subgroup was small. Longer trastuzumab was also superior to 6 months in patients that received concurrent trastuzumab. Since administration of concurrent trastuzumab and omission of anthracyclines is the most pursued strategy to date, it is likely that a longer duration might be significantly superior for most of our patients, although this is a subgroup analysis and hence underpowered to draw any conclusion. The PERSEPHONE trial started in 2007, when administration of concurrent trastuzumab plus chemotherapy was not yet the established standard in Europe, and 53% of the enrolled patients received trastuzumab after chemotherapy. This modality administration is obsolete and generated results are not informative to current treatments. Of note, the percentage of patients receiving concurrent trastuzumab increased over the years; thus results evaluated after a longer follow-up might reveal an impact of trastuzumab duration on outcomes. For overall survival analysis, heterogeneity was shown again for concurrent versus sequential trastuzumab, and for ER status, with ER-negative patients deriving a significantly higher benefit from 12-month compared with 6-month trastuzumab. This finding is in line with the higher risk of recurrence harbored by this subgroup of patients. Of note, this subgroup analysis was reported in the original manuscript but not replicated in the meta-analysis presented at ESMO 2021.^7^

### Defining high- and low-risk patients

If the benefit of trastuzumab is relatively smaller in low-risk patients, it might be argued that a shorter duration may be considered for these patients. Interestingly, the authors of the PHARE trial published a subgroup analysis assessing the 3-year benefit ratio by risk factors in terms of metastasis-free survival (MFS).^15^ Patients at very low risk (T1N0) had an excellent prognosis in both treatment arms, with no difference in terms of 3-year MFS (96.3% in both groups). Patients at low risk (T2N0 or T1N1) also did very well, with a minimal absolute difference of 1.6% between the 6- and 12-month groups (95.8 versus 94.2%). On the other hand, in patients at intermediate and high risk, the prognosis was poorer and the difference between arms, higher (3-year MFS 4.7% and 3.6%, respectively). Importantly, these subgroup analyses were not preplanned and the power in each subgroup was limited by the small sample size and number of events, although results for low-risk patients are noteworthy. It would be of interest to understand whether other prognostic biomarkers and tools might be more effective in identifying low-risk HER2-positive eBC patients for treatment de-escalation. For instance, a high level of tumor-infiltrating lymphocytes correlates with good prognosis,^16^ whereas HER2-enriched tumors by gene expression profiling are characterized by optimal response to anti-HER2 therapy.^17^ Interestingly, these two features have been recently combined along with other clinicopathological and genomic variables in a new prognostic tool called HER2DX, developed and validated on patients enrolled in the Short-HER trial. Noteworthily, HER2DX showed to predict distant MFS with more accuracy than single variables in both the internal testing and external validation cohorts,^18^ although its utility in selecting patients for treatment de-escalation needs to be prospectively validated yet.

### Alternative de-escalation strategies for HER2-positive eBC

Despite the remarkable results in patients with low-risk HER2+ eBC, it is questionable whether reducing trastuzumab duration is the most appealing and appropriate way of de-escalating adjuvant therapy, at least in countries without economic constraints. All the aforementioned trials administered a ‘strong’ chemotherapy backbone consisting of two to four drugs, including anthracyclines for most patients. Data from the APT^19^ and the ATEMPT^20^ trials showed indeed how de-escalation of chemotherapy backbone with anthracycline-free regimens is a valuable option with excellent outcomes, capable of significantly sparing toxicity. Shorter trastuzumab duration was shown to reduce the incidence of cardiac toxicities across all trials, but incidence of serious and irreversible cardiac events was very low in both arms.^9^, ^10^, ^11^^,^^21^^,^^22^ Moreover, the risk of cardiac toxicity is way lower with anthracycline-free regimens, and for these patients a shorter duration of trastuzumab is unlikely to have a meaningful impact in terms of further reduction of cardiac events. Thus far, all these de-escalated chemotherapy regimens have been investigated with 12-month trastuzumab only, and we do not have data about the efficacy and toxicity of these regimens with 6-month trastuzumab. It is noteworthy that many other efforts are ongoing to identify the optimal way to de-escalate therapy without compromising outcomes, such as the COMPASSHER2-pCR (NCT04266249), DECRESCENDO (NCT04675827), WSG-ADAPT^23^ or PHERGain^24^ trials, but all of them are focusing on chemotherapy de-escalation instead of reducing the duration of adjuvant trastuzumab. Well-powered randomized clinical trials are crucial in the context of treatment de-escalation in order to minimize caveats as well as the risk of undertreating patients.

### Conclusion

In conclusion, considering the increasing use of non-anthracycline-based regimens for HER2-positive eBC and the resultant decreased risk of cardiac toxicity, we believe that available evidence does not conclusively support reducing the duration of adjuvant trastuzumab, and 1-year treatment should remain the standard of care. Nevertheless, it is undeniable that a shorter duration might be a valuable option for patients developing cardiac toxicity, as well as for patients at low risk of relapse treated in resource-constrained countries. One year of trastuzumab showed indeed not to be cost-effective in some of these countries,^25^^,^^26^ although the availability of biosimilars could drastically cut the costs without the need for reducing treatment duration.

## References

[1] Romond E.H., Perez E.A., Bryant J. (2005). Trastuzumab plus adjuvant chemotherapy for operable HER2-positive breast cancer. N Engl J Med.

[2] Piccart-Gebhart M.J., Procter M., Leyland-Jones B. (2005). Trastuzumab after adjuvant chemotherapy in HER2-positive breast cancer. N Engl J Med.

[3] Slamon D.J. (2005). Phase III randomized trial comparing doxorubicin and cyclophosphamide followed by docetaxel (AC-> T) with doxorubicin and cyclophosphamide followed by docetaxel and trastuzumab (AC-> TH) with docetaxel, carboplatin and trastuzumab (TCH) in HER2 positive early breast cancer patients: BCIRG 006 study. Breast Cancer Res Treat.

[4] Piccart M., Procter M., Fumagalli D. (2021). Adjuvant pertuzumab and trastuzumab in early HER2-positive breast cancer in the APHINITY trial: 6 years’ follow-up. J Clin Oncol.

[5] Goldhirsch A., Gelber R.D., Piccart-Gebhart M.J. (2013). 2 years versus 1 year of adjuvant trastuzumab for HER2-positive breast cancer (HERA): an open-label, randomised controlled trial. Lancet.

[6] Earl H.M., Hiller L., Vallier A.L. (2019). 6 versus 12 months of adjuvant trastuzumab for HER2-positive early breast cancer (PERSEPHONE): 4-year disease-free survival results of a randomised phase 3 non-inferiority trial. Lancet.

[7] Earl H.M., Hiller L., Dunn J.A. (2021). Individual patient data meta-analysis of 5 non-inferiority RCTs of reduced duration single agent adjuvant trastuzumab in the treatment of HER2 positive early breast cancer. Ann Oncol.

[8] Joensuu H., Bono P., Kataja V. (2009). Fluorouracil, epirubicin, and cyclophosphamide with either docetaxel or vinorelbine, with or without trastuzumab, as adjuvant treatments of breast cancer: final results of the FinHer trial. J Clin Oncol.

[9] Conte P., Frassoldati A., Bisagni G. (2018). Nine weeks versus 1 year adjuvant trastuzumab in combination with chemotherapy: final results of the phase III randomized Short-HER study. Ann Oncol.

[10] Joensuu H., Fraser J., Wildiers H. (2018). Effect of adjuvant trastuzumab for a duration of 9 weeks vs 1 year with concomitant chemotherapy for early human epidermal growth factor receptor 2-positive breast cancer: the SOLD randomized clinical trial. JAMA Oncol.

[11] Mavroudis D., Saloustros E., Malamos N. (2015). Six versus 12 months of adjuvant trastuzumab in combination with dose-dense chemotherapy for women with HER2-positive breast cancer: a multicenter randomized study by the Hellenic Oncology Research Group (HORG). Ann Oncol.

[12] Pivot X., Romieu G., Debled M. (2019). 6 months versus 12 months of adjuvant trastuzumab in early breast cancer (PHARE): final analysis of a multicentre, open-label, phase 3 randomised trial. Lancet.

[13] Slamon D., Eiermann W., Robert N. (2011). Adjuvant trastuzumab in HER2-positive breast cancer. N Engl J Med.

[14] Perez E.A., Romond E.H., Suman V.J. (2014). Trastuzumab plus adjuvant chemotherapy for human epidermal growth factor receptor 2 - positive breast cancer: planned joint analysis of overall survival from NSABP B-31 and NCCTG N9831. J. Clin. Oncol.

[15] Kramar A., Bachelot T., Madrange N. (2014). Trastuzumab duration effects within patient prognostic subgroups in the PHARE trial. Ann Oncol.

[16] Denkert C., von Minckwitz G., Darb-Esfahani S. (2018). Tumour-infiltrating lymphocytes and prognosis in different subtypes of breast cancer: a pooled analysis of 3771 patients treated with neoadjuvant therapy. Lancet Oncol.

[17] Schettini F., Pascual T., Conte B. (2020). HER2-enriched subtype and pathological complete response in HER2-positive breast cancer: a systematic review and meta-analysis. Cancer Treat Rev.

[18] Prat A., Guarneri V., Paré L. (2020). A multivariable prognostic score to guide systemic therapy in early-stage HER2-positive breast cancer: a retrospective study with an external evaluation. Lancet Oncol.

[19] Tolaney S.M., Barry W.T., Dang C.T. (2015). Adjuvant paclitaxel and trastuzumab for node-negative, HER2-positive breast cancer. N Engl J Med.

[20] Tolaney S.M., Tayob N., Dang C. (2021). Adjuvant trastuzumab emtansine versus paclitaxel in combination with trastuzumab for stage I HER2-positive breast cancer (ATEMPT): a randomized clinical trial. J Clin Oncol.

[21] Pivot X., Suter T., Nabholtz J.M. (2015). Cardiac toxicity events in the PHARE trial, an adjuvant trastuzumab randomised phase III study. Eur J Cancer.

[22] Earl H.M., Vallier A.L., Dunn J. (2016). Trastuzumab-associated cardiac events in the Persephone trial. Br J Cancer.

[23] Nitz U.A., Gluz O., Christgen M. (2017). De-escalation strategies in HER2-positive early breast cancer (EBC): final analysis of the WSG-ADAPT HER2+/HR− phase II trial: efficacy, safety, and predictive markers for 12 weeks of neoadjuvant dual blockade with trastuzumab and pertuzumab ± weekly paclitaxel. Ann Oncol.

[24] Pérez-García J.M., Gebhart G., Ruiz Borrego M. (2021). Chemotherapy de-escalation using an 18F-FDG-PET-based pathological response-adapted strategy in patients with HER2-positive early breast cancer (PHERGain): a multicentre, randomised, open-label, non-comparative, phase 2 trial. Lancet Oncol.

[25] Gupta N., Verma R.K., Gupta S., Prinja S. (2020). Cost effectiveness of trastuzumab for management of breast cancer in India. JCO Glob Oncol.

[26] Gershon N., Berchenko Y., Hall P.S., Goldstein D.A. (2019). Cost effectiveness and affordability of trastuzumab in sub-Saharan Africa for early stage *HER2*-positive breast cancer. Cost Eff Resour Alloc.

